# Potential Significance of Serum Autoantibodies to Endometrial Antigens, α-Enolase and Hormones in Non-Invasive Diagnosis and Pathogenesis of Endometriosis

**DOI:** 10.3390/ijms242115578

**Published:** 2023-10-25

**Authors:** Irina V. Menzhinskaya, Stanislav V. Pavlovich, Arika G. Melkumyan, Vladimir D. Chuprynin, Ekaterina L. Yarotskaya, Gennady T. Sukhikh

**Affiliations:** 1 National Medical Research Center for Obstetrics, Gynecology and Perinatology Named after Academician V.I. Kulakov of the Ministry of Health of the Russian Federation, 117997 Moscow, Russia; 2 Department of Obstetrics, Gynecology, Perinatology and Reproductology, Institute of Professional Education, I.M. Sechenov First Moscow State Medical University of the Ministry of Health of the Russian Federation (Sechenov University), 119048 Moscow, Russia

**Keywords:** endometriosis, ovarian endometrioma, deep infiltrative endometriosis, autoantibodies to tropomyosin, tropomodulin, α-enolase, estradiol, progesterone, human chorionic gonadotropin

## Abstract

The objective of the study was to evaluate the profile of serum autoantibodies and their diagnostic and pathogenetic significance in ovarian endometrioma (OEM) and deep infiltrative endometriosis (DIE). The study enrolled 74 patients with endometriosis (Group 1), including 53 patients with OEM (Subgroup 1a); 21 patients with DIE without ovarian lesions (Subgroup 1b); and 27 patients without endometriosis (Group 2). The diagnosis was confirmed by laparoscopic surgery and histologic examination of resected tissues. Antibodies (M, G) to tropomyosin 3 (TPM), tropomodulin 3 (TMOD), α-enolase (ENO), estradiol (E2), progesterone (PG), and human chorionic gonadotropin (hCG) were identified in blood serum using modified ELISA. In endometriosis, antibodies to endometrial antigens, hormones, and ENO were detected more often than antiphospholipid and antinuclear antibodies. Higher levels of IgM to TPM, hCG, E2, and PG and IgG to TMOD, ENO, E2, and hCG were found in Subgroup 1a compared to Group 2. IgM to TPM, hCG, E2, PG, and IgG to E2 and ENO had a high diagnostic value for OEM (AUC > 0.7), with antibodies to TPM having the highest sensitivity and specificity (73.6% and 81.5%). In Subgroup 1b, only the levels of IgM to TPM and hCG were higher than in Group 2. These antibodies had a high diagnostic value for DIE. Thus, endometriosis is associated with autoantibodies to endometrial antigens, α-enolase, steroid, and gonadotropic hormones. A wider spectrum of antibodies is detected in OEM than in DIE. These antibodies have a high diagnostic value for OEM and DIE and potential pathogenetic significance for endometriosis and associated infertility.

## 1. Introduction

Endometriosis is a benign estrogen-dependent chronic systemic inflammatory gynecological condition with multifactorial etiopathogenesis. The disease is characterized by the presence of actively functioning foci of endometrium (glandular cells and stroma) or endometrial tissue outside the uterine cavity, i.e., in the muscular layer of the uterine wall or in the other organs of reproductive system, or/and in the adjacent or distant structures [1,2,3]. Being a common gynecologic disease, endometriosis occurs in 10–15% of women of reproductive age, including 35–50% of women with pelvic pain and/or infertility [1]. In addition, the peak incidence of endometriosis occurs in patients at the age of 25–45 years. The incidence of ovarian endometrioma (OEM) in women of reproductive age with diagnosed endometriosis reaches 55% [4].

Endometriosis often leads to chronic pelvic pain, dysmenorrhea, dyspareunia, infertility, and impaired quality of life, significantly burdening health in general [2,3], as confirmed by reliable special questionnaires [5], and represents a serious medical and socioeconomic problem. The lack of specific signs and symptoms makes accurate diagnosis a major challenge. At the same time, 45–50% of patients with endometriosis are asymptomatic. Due to this fact, the onset of treatment is delayed on average by 8–10 years; this contributes to the development of severe forms of the disease and complications [1,6].

The subtypes and localization of endometriotic lesions are diverse, including superficial peritoneal endometriosis, ovarian endometrioma, deep infiltrative endometriosis (DIE), bowel, bladder, extra-abdominal, iatrogenic endometriosis, and peritoneal adhesions [3,7]. The three most frequent forms of endometriosis are peritoneal endometriosis, ovarian endometrioma, and deep infiltrative endometriosis with the involvement of bowel or recto-vaginal septum [2].

The gold standard for the diagnosis of endometriosis is laparoscopic identification of endometriotic lesions with subsequent confirmation by histologic examination, but this invasive procedure has several limitations [8]. Among non-invasive diagnostic techniques, transvaginal ultrasound (US) is the main tool in cases of ovarian and deep endometriosis, and its accuracy is comparable to that of diagnostic laparoscopy [9]. Magnetic resonance imaging may also be useful. However, the use of these techniques is limited in the early stages and minor forms of endometriosis [8].

A number of biomarkers has been proposed for non-invasive diagnosis of endometriosis, i.e., cancer antigen-125 (CA-125), cancer antigen-19-9 (CA-19-9), interleukin-6 (IL-6), anti-endometrial antibodies (AEA); however, all of them are associated with advanced endometriosis and not applicable to the early stages of the disease [3,10]. Therefore, the biomarkers for effective early diagnosis and monitoring of endometriosis treatment are being vigorously searched.

Of great scientific interest are studies of the complex etiopathogenesis of endometriosis, involving genetic, immunological, and environmental factors. Key pathogenetic mechanisms include the inflammatory immune response and dysregulation of immune surveillance, which promotes the growth of ectopic endometrial foci [3,11,12]. It was shown that in the peritoneal fluid of patients, the number of activated macrophages with a reduced ability for phagocytosis, as well as their secretion of pro-inflammatory cytokines and prostaglandins increases [2,3], while anti-inflammatory cytokines production in stromal, epithelial, and immune cells decreases, contributing to the progression of endometriosis [13]. Moreover, there is an imbalance between type 1 (Th1) and type 2 (Th2) helper lymphocytes with a shift towards a Th2 response, in which the cytokines involved in the differentiation of B lymphocytes, suppression of cellular reactions, and enhancement of humoral reactions, are secreted [14].

Several studies in endometriosis patients have demonstrated decreased activity of cells that inhibit endometrial implantation, such as natural killer (NK) cells and CD4+/CD8+ T lymphocytes. There is also an increase in immunosuppressor cells, particularly regulatory T lymphocytes, Th2 cells, and MDSCs (suppressor cells of myeloid origin), which may contribute to endometrial cell implantation and endometriosis progression [12].

Endometriosis has overlapping similarities with autoimmune diseases such as polyclonal B-cell activation, abnormalities in T- and B-lymphocyte function, high levels of cytokines, clinical response to immunomodulators, involvement and damage to many organs and tissues, hereditary and family nature of the disease, and combination with other autoimmune disorders [15]. Endometriosis is often associated with a number of autoimmune diseases including systemic lupus erythematosus, fibromyalgia, Sjögren’s syndrome, inflammatory bowel disease, and others, which are more prevalent in patients with endometriosis than in the general population [16,17]. The dysregulation of the immune system observed in autoimmune diseases leads to changes in cell-mediated and humoral immunity, which contribute to the development of endometriosis. In turn, concomitant autoimmune disorders cause a more severe course of endometriosis [18].

A large number of studies hypothesize the involvement of B-lymphocytes producing anti-endometrial (AEA), antiphospholipid (aPL), antinuclear (ANA), and anti-DNA autoantibodies, typical for other autoimmune diseases, in the pathogenesis of endometriosis [15,19]. In addition, endometriosis-associated endocrine disorders, including increased production of steroid hormones, contribute to the development of autoimmune processes [20].

Using a two-dimensional proteomic approach to study the serum of endometriosis patients positive for AEA, target endometrial antigens were identified in the range from 30 kDa to 45 kDa [21], particularly, tropomyosin 3 (TPM) and tropomodulin 3 (TMOD), which may be involved in cytoskeletal protein functions such as proliferation, apoptosis, cell motility, and adhesion, as well as in the receptor and secondary messenger functions [3], while antibodies to these antigens have been proposed as biomarkers for non-invasive diagnosis of endometriosis [22].

The study of autoantibodies to molecules that play an important role in the pathophysiology of endometriosis, in particular to specific endometrial antigens (TPM, TMOD), the glycolytic enzyme α-enolase 1 (ENO) involved in endometrial cell invasion, as well as to steroids (estradiol (E2), progesterone (PG)) and gonadotropins, in various forms of endometriosis, may contribute to a deeper understanding of the immune aspects of the pathophysiology of endometriosis, identifying promising diagnostic biomarkers and proposing new treatment strategies [23]. In connection with the above, the aim of this study was to evaluate the profile of serum autoantibodies and their diagnostic and pathogenetic significance in ovarian endometrioma and deep infiltrative endometriosis.

## 2. Results

All study groups were comparable in terms of the mean age of the patients (Table 1). In Group 1, 71.6% of patients (53/74) had stage II–IV OEM and 77% (57/74) had stage III–IV DIE according to the revised American Society for Reproductive Medicine classification (rASRM). A combination of OEM and DIE was present in 48.6% (36/74) of patients, while 28.4% of patients (21/74) had DIE without ovarian lesions. Adenomyosis, pelvic adhesions, infertility, and uterine fibroids were mostly or more prevalent in Group 1 (Table 1).

In Subgroup 1a, ovarian endometriomas were combined with DIE in 67.9% of patients (36/53). In Subgroup 1b, where all patients had DIE, there was a higher incidence of bowel and bladder endometriosis and adenomyosis compared with Subgroup 1a (*p* < 0.0001; *p* = 0.022). Patients in both subgroups equally often had pelvic adhesions (*p* = 0.789). Subgroups 1a and 1b did not differ by the rates of infertility (*p* = 0.745), uterine fibroids (*p* = 0.893), as well as parity, induced abortions, and spontaneous miscarriages.

Serum levels of autoantibodies in patients with and without endometriosis are presented in Table 2. Patients with endometriosis of Subgroups 1a and 1b and patients without endometriosis of Group 2 differed in the level of IgM antibodies to TPM, PG, E2, and hCG and IgG antibodies to TMOD and E2 (*p* < 0.025). In a pairwise comparison of Subgroups 1a and 1b with Group 2, the patients with OEM had higher levels of IgM antibodies to TPM, PG, E2, and hCG and IgG antibodies to TMOD, ENO, E2, and hCG (*p* < 0.025). Notably, in Subgroup 1a, median autoantibody levels did not differ between patients with and without DIE (*p* > 0.05). At the same time, in the patients of Subgroup 2b with DIE without ovarian lesions, only increased levels of IgM antibodies to TPM and hCG were observed (*p* values <0.001 and 0.01, respectively). When comparing Subgroups 1a and 1b, patients in Subgroup 1a had higher levels of IgG antibodies to E2 and TMOD than patients in Subgroup 1b (*p* values of 0.01 and 0.02, respectively).

Increased levels of antiphospholipid and antinuclear antibodies were detected only in 2/53 (3.8%) patients with OEM.

**Figure 1:**
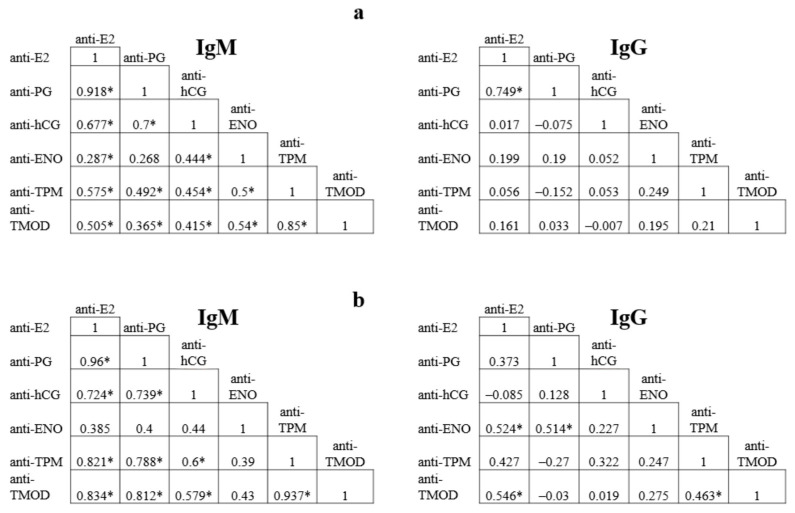
Correlation between the levels of autoantibodies (M, G) to estradiol (anti-E2), progesterone (anti-PG), human chorionic gonadotropin (anti-hCG), α-enolase (anti-ENO), tropomyosin (anti-TPM), tropomodulin (anti-TMOD) in patients with ovarian endometrioma (**a**) and deep infiltrative endometriosis without ovarian lesions (**b**). * *p* < 0.05.

In patients with OEM, a direct correlation was found between the levels of class M and G antibodies to estradiol and progesterone, and between the levels of IgM antibodies to TPM and TMOD (r values of 0.92; 0.75 and 0.85, respectively; *p* < 0.001) (Figure 1). In addition, there was a direct correlation between the levels of IgM antibodies of other specificities with r < 0.7; this correlation was more significant between the antibodies to different hormones, as well as between anti-endometrial antibodies and antibodies to ENO. No correlation was observed between the levels of other IgG antibodies.

In DIE, a direct correlation was also found between IgM antibodies to hormones E2, PG, and hCG, as well as between IgM to TPM, TMOD and E2, and PG (r > 0,7; *p* < 0,001), while r > 0.9 was obtained when comparing IgM to E2 and PG, TPM and TMOD. Moreover, a direct correlation was found between IgG antibodies to TPM and TMOD (r = 0.46; *p* = 0.04), and between IgG antibodies to E2 and ENO (r = 0.52; *p* = 0.02) or TMOD (r = 0.54; *p* = 0.01), to PG and ENO (r = 0.51; *p* = 0.02).

According to ROC analysis, six autoimmune markers (IgM antibodies to TPM, hCG, E2, and PG; IgG antibodies to ENO and E2) had high diagnostic significance for ovarian endometriomas, confirmed by high values of the area under the ROC-curve (AUC 0.712–0.848; *p* < 0.001) (Table 3). Determination of IgM antibodies to TPM, hCG, and E2 showed higher sensitivity (≥60%) for diagnosing endometrioma at specificity ≥80%. In addition, according to logistic regression analysis, the determination of IgM antibodies to TPM and hCG was characterized by high accuracy in diagnosing OEM, which was 83.8% and 75%, respectively. The number of OEM patients with levels of IgM antibodies to TPM, hCG, E2, and PG exceeding the cutoff values calculated using the ROC-analysis was 4.6–12.6 times higher than in the comparison group.

In DIE without ovarian lesions, only IgM antibodies to TPM (AUC 0.844 (0.708–0.933); *p* < 0.0001) and hCG (AUC 0.736 (0.587–0.854); *p* = 0.0013) had the high diagnostic value. According to logistic regression analysis, when determining IgM antibodies to TPM, the diagnostic accuracy for DIE was the maximum and reached 83.8%. The number of DIE patients with a level of IgM antibodies to hCG and TPM exceeding the cutoff values was 4.0 and 8.8 times greater than in the group without endometriosis.

## 3. Discussion

The present study examined the serum autoantibody profile, including anti-endometrial, antiphospholipid, antinuclear antibodies, as well as antibodies to α-enolase and hormones, namely estradiol, progesterone, and human chorionic gonadotropin, in patients with different forms of endometriosis (ovarian endometrioma and deep infiltrative endometriosis), and in the comparison group without endometriosis. It should be noted that the possible influence on the study results of systemic autoimmune, oncological, infectious, inflammatory, and endocrine diseases, metabolic syndrome, as well as the use of hormonal and anti-inflammatory drugs in patients was excluded.

The groups were formed according to laparoscopic surgery findings and histological confirmation of the diagnosis. Subgroup 1a included the patients with ovarian endometriomas, which in most cases were associated with DIE, and, less often with bowel and bladder endometriosis, as well as adenomyosis. In Subgroup 1b, bowel endometriosis and adenomyosis was found significantly more often than in Subgroup 1a. Patients of both subgroups were comparable in age and did not differ by the rates of pregnancy and childbirth, induced abortions, or spontaneous miscarriages.

Pelvic adhesions and infertility were frequently observed in both subgroups. Endometriosis-associated pelvic adhesions often result from pro-inflammatory changes in the peritoneal media and tissues. Endometriosis can cause adhesions in up to 60% of cases [24]. However, in endometriosis, inflammation is not limited to the abdominal cavity; immune responses extend to the systemic level [25].

The pathophysiology of endometriosis is not fully studied; there still are many theories about the pathogenesis of this disease. There is no consensus on whether different forms of endometriosis have a common pathogenesis, and whether one type can cause another. It is believed that deep endometriosis may be more likely to be of embryological origin; therefore, the immune system plays a minor role in its development [12]. Superficial peritoneal endometriosis and ovarian endometriomas are usually considered to be the consequences of implantation of endometrial cells coming to the pelvic cavity through the fallopian tubes with retrograde menstrual flow. In this case, impaired immune response and dysregulation of immune surveillance are critical factors, hampering the elimination of ectopic endometrium and, thus, promoting the development of endometriosis and associated conditions, including pelvic adhesions and infertility.

Endometriosis is characterized by polyclonal activation of B lymphocytes, dysfunction of T (Th1) and B lymphocytes, impaired apoptosis and NK cell activity, imbalance of Th1/Th2 and increased Th2-dependent response, induction and translocation of T regulatory cells, release of immunological and inflammatory factors such as cytokines, and increased production of autoantibodies [12,14]. Peritoneal immune responses in endometriosis may be due to the increased concentrations of IgG and IgA and altered expression of pro-inflammatory cytokines and transcription factors [26]. Expression of B lymphocyte stimulator (BLyS), necessary for B cell proliferation and differentiation, is dramatically elevated [27], leading to overactivation of B cells and excessive production of autoantibodies, and contributes to the progression of endometriosis. In addition, the expression of B-cell lymphoma 6 (BCL6) transcription factor, which is a critical regulator of humoral immunity, is increased [28].

The results of the present study demonstrated a wide spectrum of serum autoantibodies in endometriosis, including antibodies to endometrial antigens (TMOD, TPM), steroid and gonadotropic hormones (E2, PG, hCG), and α-enolase enzyme. Patients with endometriosis had higher levels of IgM antibodies to TPM, PG, E2, hCG, and of IgG antibodies to E2 and TMOD, compared to women without endometriosis. In endometriosis, these antibodies were more common than antiphospholipid and antinuclear antibodies.

Patients with OEM had a wide spectrum and higher levels of serum autoantibodies of different specificities, in particular IgM antibodies to TPM, E2, PG, and hCG, as well as IgG antibodies to TMOD, ENO, E2, and hCG, compared to women without endometriosis. At the same time, in patients with DIE without ovarian lesions, only the levels of IgM antibodies to TPM and hCG were significantly increased. In addition, the levels of IgG antibodies to E2 and TMOD were significantly higher in patients with OEM than in patients with DIE. More frequent detection of high levels of IgM antibodies to endometrial and hormonal antigens may be associated with the progressive course of endometriosis and the increased content of these proteins, especially TPM, in proliferating endometrial cells in ectopic foci and active secretion of hormones.

Our results are consistent with a systematic review [19], showing that the majority of 22 studies on the role of B lymphocytes in endometriosis had found an excessive production of antibodies and pro-inflammatory cytokines due to increased number and/or activation of B cells.

In patients with OEM, a strong direct correlation was found between the levels of IgM and IgG antibodies to E2 and PG, IgM antibodies to TPM and TMOD (r > 0.7), while weaker direct correlation was observed between IgM antibodies of other specificities (r < 0.7). In DIE, a correlation was found between IgM antibodies to hormones, including hCG, as well as to endometrial antigens and steroid hormones (r > 0.7), while the highest r value was obtained when comparing IgM levels to E2 and PG, TPM and TMOD (r >0.9). A direct correlation between the levels of these autoantibodies may result from polyclonal activation of B lymphocytes to hormonal and endometrial antigens, the levels of which are significantly increased in endometriosis. The obtained data on specific patterns of correlation expand the understanding of the pathophysiology of endometriosis and may be useful in creating a diagnostic panel of autoantibodies.

The results of the present study are consistent with the data of R. Gajbhiye et al., who revealed a high rate (60%) of anti-endometrial antibodies (M, G) detection in patients with endometriosis, including OEM [29]. Later, R. Gajbhiye et al. showed the high diagnostic accuracy of new biomarkers, particularly, the antibodies to various TPM and TMOD epitopes, for early stages of endometriosis (I–II), with the sensitivity of 52–68% and specificity ≥80%, which were higher than those of the CA-125 (24% and 89%, respectively) [22]. The authors identified antibodies to six TMP and TMOD epitopes that had sensitivity ≥63% and specificity ≥80% for stage 1–2 and US-negative endometriosis; these parameters did not differ significantly in the follicular and luteal phases of the menstrual cycle. With the use of the panel of six biomarkers proposed for early diagnosis of endometriosis, the sensitivity increased to 79% and specificity to 80%, and these parameters exceeded those of serum CA 19-9 (43% and 48%), α-enolase (36% and 80%), and syntaxin 5 (38% and 80%).

Anti-endometrial antibodies studied by R. Gajbhiye et al. [22] had showed higher sensitivity (63–76%) and specificity (≥80%) for the diagnosis of US-negative endometriosis than CA-125 (76% and 60%), CA-19-19 (73% and 56%), intercellular adhesion molecule (sICAM)-I. (83% and 59%), and glycodelin (74% and 57%) in the study by A. Vodolazkaia et al. [30].

In the present study, we examined the blood sera of patients with OEM and DIE stages II–IV, collected during the follicular phase of the menstrual cycle, using modified ELISA methods. Three types of autoantibodies of different specificity have been identified, including not only anti-endometrial (IgM to TPM), but also anti-hormonal antibodies (IgM to hCG and E2), which have shown high sensitivity (≥60%) and specificity (≥80%) and accuracy (up to 83.8%) for OEM diagnosis. At the same time, IgM antibodies to PG and IgG antibodies to E2 and ENO showed sensitivity from 52.8% to 58.2% and specificity >80% for OEM. Notably, only IgM antibodies to TPM and hCG showed a high diagnostic value for DIE without ovarian lesions: sensitivity 70% and 50%, specificity 81.5% and 85.2%, respectively. Of particular note is the high diagnostic value of antibodies to TPM in both forms of endometriosis. In addition to anti-endometrial antibodies, in our opinion, antibodies to steroid and gonadotropic hormones can be proposed as biomarkers of endometriosis.

The autoantibodies are believed to be involved in the pathophysiology and development of endometriosis by stimulating the immune system and maintaining inflammation. Autoantibodies can bind to cell surface antigens and form immune complexes with soluble antigens, thus enhancing inflammation and tissue damage by recruiting neutrophils and other myeloid and lymphoid cells to the lesions [31]. Another mechanism involves activation of a classical complement pathway through the interaction of the C1q component with the Fc domains of antibodies in immune complexes; activation of immune cells, including effector cells with Fcγ receptors on the surface, such as monocytes, neutrophils, macrophages, dendritic cells, and natural killer cells; and an increase in production of pro-inflammatory cytokines. In turn, increased cytokine levels in endometriotic foci, caused by inflammation, stimulate the production of autoantibodies by B lymphocytes.

While immune infiltration and a persistent inflammatory environment favor the development of endometriosis, the greatest pathological consequences arise from aberrant complement activation [32]. Thus, a recent study showed dysregulation and activation of the complement system, both classical and lectin pathways, and significantly higher concentrations of C1q, MBL, and C1-INH in the peritoneal fluid of women with endometriosis compared with controls [33]. Apparently, the detected increased levels of autoantibodies can lead to activation of the classical complement pathway.

Production of autoantibodies to gonadotropic and steroid hormones in endometriosis may be associated with endocrine disorders characteristic of endometriosis, leading to increased levels of hormones in the blood, as well as with the use of hormonal therapy. Intensive production of antibodies to steroid hormones in patients with endometriosis may be promoted by excessive production of estrogens in the ovaries, ectopic endometrium, peripheral adipose tissue, and by increased production of progesterone by stromal cells in endometriotic heterotopia [34]. In addition, low expression of 17β-Hydroxysteroid dehydrogenases type 2 (17βHSD) enzyme, which converts estradiol (E2) to estrone (E1), increases E2 level [35]. Local biosynthesis of estradiol in endometriotic lesions on the background of increased inflammation in the peritoneal cavity creates an abnormal immune-endocrine microenvironment, which promotes cells growth and survival in ectopic lesions.

Endometriosis is characterized by altered PG signaling in the endometrium, which disrupts decidualization and leads to the development of ectopic lesions [36]. It is known that PG is involved in suppression of inflammation in the endometrium; therefore, inadequate PG signaling makes up a pro-inflammatory phenotype. The resulting resistance to PG is a key endometrial factor in the pathogenesis of endometriosis-associated infertility, and also a factor that makes hormone therapy ineffective in some patients. An increase in the level of antibodies to PG, detected in patients with endometriosis in the present study, suggests their involvement in the pathophysiology of endometriosis, the development of endometrial resistance to PG, and, as a consequence, a decrease in the effectiveness of hormonal therapy in seropositive patients.

Of special interest is the detection of antibodies to endometrial antigens (TPM, TMOD) and ENO, which are involved in important mechanisms of endometriosis pathogenesis associated with cell motility and migration, adhesion and invasion at the ectopic sites, cytoskeletal dynamics, conversion to stationary state, apoptosis, and necrosis [37,38,39]. In addition, the glycolytic enzyme α-enolase facilitates endometrial cells invasion by promoting plasmin activation and degradation of extracellular matrix.

The increased expression of these molecules in endometriotic cells seems to stimulate the formation of relevant antibodies. This assumption is supported by the study of the proteins of endometrial tissue lysates obtained from superficial and deep endometriotic lesions [40], which demonstrated different expression of numerous proteins involved in the implantation of cells outside the uterine cavity and involved in the progression of the disease. In particular, an increased expression of TPM in ectopic endometrial tissue, compared to eutopic tissue, became even higher in the secretory phase. An increased expression of TPM in deep endometriosis, probably due to a higher content of smooth muscle cells in the lesions, was also shown. This can explain our finding of significant increase of anti-TMP antibody levels in patients with DIE.

Altered cellular and humoral immune responses in endometriosis, including increased production of pro-inflammatory cytokines and autoantibodies, can potentially affect women’s fertility. High levels of certain autoantibodies, especially anti-endometrial and anti-hormonal, can lead to a decrease in endometrial receptivity and the quality of oocytes and embryos and impaired embryo implantation and finally result in subfertility and even infertility. Therefore, screening for the mentioned autoantibodies, in addition to antiphospholipid, antinuclear, antithyroid, and antiovarian antibodies, seems to be a necessary part of investigation of endometriosis-associated infertility.

The limitations of present investigation were: small size of the study and control groups, restricted by laparoscopic confirmation of the diagnosis; the absence of patients with stage I endometriosis in the study group; the presence of gynecological and somatic diseases in patients of the comparison group. Moreover, not all endogenous mechanisms triggering autoantibody production and not all possible confounders were taken into account in this study.

## 4. Materials and Methods

### 4.1. Ethics and Sample Collection

This study project was approved by the Ethics Committee of the National Medical Research Center for Obstetrics, Gynecology, and Perinatology. The patients signed informed consent to participate in the study and to have their biological material tested. All patients underwent laparoscopic surgery in the Department of Surgery of the National Medical Research Center for Obstetrics, Gynecology and Perinatology between January 2019 and June 2021.

The study group (Group 1) included 74 women with endometriosis aged 20 to 40 years. Endometriosis was diagnosed in all patients by laparoscopic identification of endometriotic lesions and subsequent histopathologic examination of the resected tissues. All patients with endometriosis were categorized by stage according to the rASRM. The study group was divided into two subgroups: Subgroup 1a comprised 53 patients with stage II–IV OEM, Subgroup 1b comprised 21 patients with stage III–IV DIE without ovarian lesions. The comparison group (Group 2) included 27 women with no endometriosis at diagnostic laparoscopy.

Patients with oncologic and systemic autoimmune diseases and contraindications for surgical treatment were not included in the study. Exclusion criteria were: infectious, inflammatory, endocrine diseases, metabolic syndrome, hormonal and anti-inflammatory therapy during the last three months before the study.

Blood for serum autoantibody profile evaluation was collected from women in the follicular phase of the menstrual cycle on day 10.2 ± 3.2 after fasting and immediately before laparoscopic surgery on an empty stomach. Blood serum samples were processed according to standard operating procedure, including centrifugation at 3000 rpm for 10 min at 4 °C, and stored at −80 °C in the centralized Biobank of the National Medical Research Center for Obstetrics, Gynecology, and Perinatology until testing within 2.5 years after collection. The entire set of samples was examined simultaneously and was not subjected to repeated freeze-thaw cycles.

### 4.2. ELISA for Detection of Serum Autoantibodies

Detection of antibodies (M, G) to steroid hormones, namely progesterone (PG) and estradiol (E2), was performed by means of modified ELISA described previously [41], using Thermo Scientific™ Nunc™ MaxiSorp microplates (Thermo Fisher Scientific Inc., Waltham, MA, USA), conjugates β-estradiol 6-(O-carboxymethyl)oxime: BSA (Sigma-Aldrich, Burlington, VT, USA), and progesterone 3-(O-carboxymethyl)oxime: BSA (XEMA LLC, Moscow, Russia). To block nonspecific binding sites, instead of a mixture of 1.5% (weight/volume [*w*/*v*]) bovin serum albumin (BSA) and 1.5% *w*/*v* gelatin in Tris-buffered solution, pH 7.4, the 1% *w*/*v* BSA in 100 mmol/L phosphate-buffered saline (PBS), pH 7.4, was used and incubated for 1.5 h at room temperature (20 ± 2 °C). The blood serum samples were analyzed at a 1:100 dilution in 0.5% (*w/v*) BSA-PBS containing 0.05% (*v*/*v*) Tween-20 in the duplicate, incubated at room temperature on a shaker at 200 rpm for 1 h. Antibodies (M, G) to chorionic gonadotropin were analyzed by ELISA method described elsewhere [42], using a highly purified preparation of hCG (Sigma-Aldrich, Burlington, VT, USA). Conjugates of monoclonal antibodies against human immunoglobulins M and G with horseradish peroxidase, ELISA reagents and buffers of XEMA LLC (Moscow, Russia) were used in these assays.

Antibodies to endometrial antigens (TPM, TMOD) and ENO enzyme were identified by modified ELISA methods described elsewhere [19,43], with the use of recombinant human proteins Tropomyosin 3, Tropomodulin 3, and α-Enolase 1 (Abcam PLC, Cambridge, UK) immobilized on Thermo Scientific™ Nunc™ MaxiSorp microplates (Thermo Fisher Scientific Inc., Waltham, MA, USA) at a concentration of 2–5 µg/mL. To block nonspecific binding sites, 1% *w*/*v* BSA in 100 mmol/L PBS, pH 7.4, was used instead of 1% *w*/*v* gelatin. Serum samples diluted 1:100 were incubated at room temperature on a shaker for 1 h as described above. Optical density (OD) was measured on an Infinite F50 automated enzyme immunoassay analyzer (TECAN Austria GmbH, Grödig, Austria) at a wavelength of 450 nm. The inter- and intra-assay variances in these ELISA methods were <15% and <10%, respectively.

Antiphospholipid antibodies (aPL) of M and G classes to cardiolipin (CL) and β2-glycoprotein-I (β2-GP-I) and antinuclear IgG antibodies (ANA) were detected in the serum using immunoenzyme kits (ORGENTEC Diagnostika GmbH, Mainz, Germany).

### 4.3. Statistical Analysis

The obtained data were analyzed using applied statistical software packages Microsoft Office Excel 2010 (14) and Statistica 10 (StatSoft Inc., Tulsa, OK, USA). Normality of distribution of values in the samples was assessed using the Shapiro–Wilk W-test and the Kolmogorov–Smirnov test. In case of deviation from normal distribution, quantitative data were represented by median value (Me) with interquartile range between 25th percentile (Q1) and 75th percentile (Q3). Quantitative data were analyzed using non-parametric statistical methods. Data in two groups were compared using Mann–Whitney U-test, while in three groups the data were compared using Kruskal–Wallis test and Bonferroni correction for multiple comparisons. Qualitative data were represented by absolute (n) and relative values (%); the differences were evaluated using the χ2 test. Correlation between the variables was assessed by calculating the Spearman’s Rank correlation coefficient. Differences were considered statistically significant at *p* < 0.05.

## 5. Conclusions

The results of this study demonstrated that endometriosis is associated with the presence of serum autoantibodies to endometrial antigens, α-enolase, and to steroid and gonadotropic hormones, showing different autoantibody profiles in ovarian endometrioma and deep infiltrative endometriosis. Anti-endometrial and anti-hormonal autoantibodies have high diagnostic value for these forms of endometriosis, and also a potential pathogenetic significance in the progression of endometriosis and the development of endometriosis-associated infertility.

We believe that further studies involving large cohorts of patients with various forms and early stages of endometriosis, and women without the disease, are required to extend obtained data, to investigate the dynamics of the levels of autoantibodies after treatment and during the relapse of the disease, and to evaluate the effectiveness of new methods of treatment, including immunotherapy.

## Figures and Tables

**Table 1:** Demographic and clinical characteristics of patients with different forms of endometriosis and without endometriosis.

Parameter	Group 1 Endometriosis		Group 2 Non-Endometriosis n = 27	*p*-Value ***
	Subgroup 1a OEM n = 53	Subgroup 1b DIE n = 21		
Age, years *	31 (20; 38)	33 (23; 38)	30 (21; 39)	0.21
Ovarian endometriomas **	53 (100%)	0 (0%)	0 (0%)	<0.001
Deep infiltrating endometriosis **	36 (67.9%)	21 (100%)	0 (0%)	<0.001
Bowel endometriosis **	7 (13.2%)	18 (85.7%)	0 (0%)	<0.001
Bladder endometriosis **	2 (3.8%)	2 (9.5%)	0 (0%)	0.24
Adenomyosis **	11 (20.8%)	10 (47.6%)	0 (0%)	<0.001
Pelvic adhesions **	22 (41.5%)	8 (38.1%)	0 (0%)	<0.001
Infertility	30 (56.6%)	11 (52.4%)	2 (7.4%)	0.0001
Uterine fibroids	16 (30.2%)	6 (28.6%)	3 (11.1%)	0.157

Note: *—Me (min; max), Kruskal–Wallis test; **—n (%), ꭓ2-test; ***—when comparing Subgroups 1a and 1b to Group 2, differences are significant with Bonferroni correction at *p* < 0.025.

**Table 2:** Autoantibody levels (OD units) in blood serum of the patients with ovarian endometrioma, with deep infiltrative endometriosis, and without endometriosis.

Parameter	Group 1 Endometriosis		Group 2 Non-Endometriosis n = 27	*p*-Value **
	Subgroup 1a, OEM n = 53	Subgroup 1b DIE n = 21		
anti-TPM IgG	0.273 (0.143; 0.273) *	0.307 (0.144; 0.577)	0.220 (0.130; 0.474)	0.11
anti-TPM IgM	0.278 (0.144; 0.765)	0.247 (0.161; 0.700)	0.160 (0.116; 0.540)	<0.001
anti-TMOD IgG	0.306 (0.120; 0.907)	0.232 (0.134; 0.650)	0.210 (0.132; 0.636)	0.01
anti-TMOD IgM	0.222 (0.118; 0.630)	0.192 (0.130; 0.542)	0.209 (0.106; 0.650)	0.89
anti-ENO IgG	0.305 (0.167; 0.777)	0.279 (0.177; 0.606)	0.215 (0.111; 0.385)	0.046
anti-ENO IgM	0.235 (0.113; 0.990)	0.285 (0.153; 0.640)	0.280 (0.146; 0.547)	0.07
anti-PG IgG	0.260 (0.124; 1.48)	0.198 (0.134; 0.471)	0.256 (0.157; 0.447)	0.08
anti-PG IgM	0.350 (0.154; 0.891)	0.241 (0.164; 0.871)	0.212 (0.146; 0.466)	<0.001
anti-E2 IgG	0.346 (0.129; 1.19)	0.249 (0.174; 0.513)	0.261 (0.154; 0.572)	0.002
anti-E2 IgM	0.318 (0.098; 0.772)	0.246 (0.154; 0.838)	0.220 (0.115; 0.495)	0.001
anti-hCG IgG	0.262 (0.183; 0.925)	0.219 (0.150; 0.683)	0.229 (0.136; 0.421)	0.047
anti-hCG IgM	0.240 (0.133; 0.494)	0.217 (0.155; 0.467)	0.172 (0.122; 0.362)	<0.001

Note: *—Me (min; max), OD units; **—Kruskal–Wallis test; differences are significant with Bonferroni correction at *p* < 0.025. Abbreviation: TPM, tropomyosin; TMOD, tropomodulin; ENO, α-enolase; hCG, human chorionic gonadotropin; E2, estradiol; PG, progesterone.

**Table 3:** Diagnostic value of serum autoantibodies for ovarian endometrioma and deep infiltrative endometriosis without ovarian lesions according to ROC- analysis.

Parameter	Cutoff (OD Units)	Se * (%)	Sp (%)	AUC (95% CI)	Accuracy ** (%)	DOR	*p*-Value
Ovarian endometrioma (n = 53)							
Anti-TPM IgM	≥0.214	73.6	81.5	0.848 (0.750–0.918)	83.8	12.6	<0.0001
Anti-hCG IgM	≥0.211	64.2	81.5	0.798 (0.693–0.879)	75	7.3	<0.0001
Anti-E2 IgM	≥0.268	64.2	81.5	0.769 (0.661–0.856)	66.3	6.3	<0.0001
Anti-PG IgM	≥0.287	58.5	81.5	0.766 (0.658–0.853)	67.5	4.6	<0.0001
Anti-ENO IgG	≥0.297	52.8	81.5	0.712 (0.600–0.808)	68.8	4.9	<0.0001
Anti-E2 IgG	≥0.332	52.8	85.2	0.717 (0.605–0.812)	70	4.9	0.0003
Deep infiltrative endometriosis (n = 21)							
Anti-TPM IgM	≥0.214	70	81.5	0.844 (0.708–0.933)	83.8	8.8	<0.0001
Anti-hCG IgM	≥0.213	50	85.2	0.736 (0.587–0.854)	70.2	4.0	0.0013

Note: *—maximum sensitivity at specificity of ≥80%; **—(true-positive cases + true-negative cases)/total number of cases × 100. Abbreviation: AUC, area under the ROC curve; CI, confidence interval; Se, sensitivity; Sp, specificity; DOR, diagnostic odds ratio; ROC, receiver-operating characteristic curve; TPM, tropomyosin; hCG, human chorionic gonadotropin; E2, estradiol; PG, progesterone; ENO, α-enolase.

## Data Availability

The data that support the findings of this study are available from the corresponding author, upon request.
